# *De novo* generation of viruses in animals: from infection models to vaccine development

**DOI:** 10.1128/msphere.00742-24

**Published:** 2025-08-11

**Authors:** Maxime Cochin, Jean-Sélim Driouich, Léa Luciani, Antoine Nougairède

**Affiliations:** 1 Unité des Virus Émergents (UVE: Aix-Marseille Univ, Università di Corsica, IRD 190, Inserm 1207, IRBA), Marseille, France; University of Michigan, Ann Arbor, Michigan, USA

**Keywords:** reverse genetics, virology, viral *de novo* generation *in vivo*, vaccines, DNA-launched live attenuated vaccines

## Abstract

Reverse genetics is a valuable research tool in the field of virology with numerous applications. Primarily employed to recover viral strains *in vitro*, these approaches have also been used to generate viral strains directly in animals. This review presents a historical and technical overview of publications describing the rescue of viruses from injection of nucleic acids directly into animals since the 1950s. The injection of purified or cloned viral genomes *in vivo* has enabled the generation of many pathogens in a wide range of animal models. Recent advances in the delivery process of nucleic acid into cells have also contributed to this field of virology research, which currently focuses on creating a new generation of vaccines called DNA-launched live attenuated vaccines (LAVs). This new approach simplifies the administration of existing or newly created LAV strains, while providing better control of the inoculated material.

## HISTORICAL OVERVIEW

### Initial discoveries

The infectious potential of RNA virus genomes when inoculated *in vivo*, especially in plants, was discovered in 1956 by Gierer and Schramm in their work on tobacco mosaic virus (1). Following this landmark discovery, other teams repeated the experiment with positive-stranded RNA viruses in mammals. Colter and Brown *de novo* generated mengovirus, poliomyelitis virus, and West Nile virus (WNV) *in vivo* from purified viral RNA inoculated intracerebrally into mice (2, 3). In these studies, inoculum degradation by ribonucleases was a common means to demonstrate the infectious capacity of genomic viral RNA alone and to dispel doubts about the presence of remaining viral particles in injected infectious material. Regarding the infectious potential of DNA virus genomes inoculated into animals, the earliest research, which dates back to the 1960s, focused on the oncogenic properties of viruses. The first work on this subject linked the occurrence of tumours in domestic rabbits to intradermal (ID) inoculation of viral DNA obtained from cottontail rabbit warts infected with a kappapapilomavirus 2 (KPV2) strain (4). The absence of tumour formation when the viral DNA was pretreated with deoxyribonuclease demonstrated the infectivity of the nucleic acids. Other purified DNA virus genomes from other DNA viruses, such as simian adenovirus (SAdV) and simian virus 40 (SV40), were also able to induce tumours after subcutaneous (SC) injection in other animal models, such as rabbits and hamsters (5–7). The work of Harris et al. demonstrated viral replication in parallel with oncogenesis after isolation of the virus from tumours induced by SC inoculation of purified genomic DNA of the Mill Hill polyomavirus into newborn ferrets (8).

In the early 1980s, viral infection models were developed on the basis of the inoculation of viral genomes that had been cloned into double-stranded DNA vectors using molecular biology techniques (9). Will et al. were the first to demonstrate that an infectious clone encoding the hepatitis B virus (HBV) induced viral hepatitis in chimpanzees after simultaneous inoculation by the intramuscular (IM), intravenous (IV), and intrahepatic routes, or by the hepatic route alone (9, 10).

In 1992, the first model demonstrating the *de novo* generation of virus *in vivo* using RNA transcripts obtained by reverse genetics methods was published (11). In this study, Emerson et al. showed that intrahepatic inoculation of transcripts produced *in vitro* from an infectious clone of hepatitis A virus (HAV) caused hepatitis and shedding of the virus in chimpanzees.

### Application to the study of viruses

Following these discoveries, numerous infection models based on the inoculation of cloned viral genomes have been developed to facilitate virology research.

In the early 1990s, in the midst of the acquired immunodeficiency syndrome pandemic, the scientific and medical research efforts on human immunodeficiency virus (HIV) led to the development of models using related retroviruses (12). Letvin et al. have shown that macaque monkeys can be infected by inoculation with an infectious simian immunodeficiency virus (SIV) DNA clone (13). Pion and Liska later developed another infectious clone to study the pathogenesis, latency, and integration of the SIV viral genome (14–16). More recently, the same type of model was used to study the attenuation of this virus (17). Regarding feline immunodeficiency virus (FIV), two models of infectious clone infection in cats were published in 1997 (18, 19). This method of infection was used in another study looking at neurological and immunological disorders associated with FIV infection (20).

Advances in knowledge toward lentiviruses and their *de novo* generation using infectious clones into animals were then used to study human T-lymphotropic virus (HTLV), another retrovirus discovered a few years before HIV (21). In order to understand the role of retrotranscription of the HTLV genome, Zhao et al. showed that DNA genome copies were infectious when inoculated into rabbits, suggesting an important role of retrotranscripts in viral latency. During the 1990s, other similar models using a related virus, bovine leukemia virus (BLV), were developed in sheep, rabbits, and rats (22–24). These new models also made it possible to study the recombination capacity of cloned viral genomes. Simultaneous inoculation of two clones deleted from different genomic regions of BLV in sheep enabled the restoration of infectious viral particles (25). This ability was already highlighted by the work of Sprengel et al. on duck HBV (DHBV) (26).

These techniques can also address the problem of “fastidious” viruses such as hepatitis C and hepatitis E viruses (HCV and HEV). They simplified the study of these viruses, for which conventional *in vitro* and *in vivo* infection models were either absent or exceedingly complicated. In the late 1990s, multiple studies demonstrated the ability of *de novo* generation of HCV in chimpanzees by injecting RNA transcripts from infectious clones (27, 28). Subsequently, the genetic determinants of HCV (29–33) and HEV (34–43) pathogenicity were investigated in numerous studies using this kind of infection model.

### A future weapon against viral infection?

The application of these techniques could potentially lead to the creation of a new generation of live attenuated vaccines (LAVs) delivered via nucleic acid form. To date, this process has only been used for RNA viruses.

Vaccination is a cornerstone of population protection against viral diseases. LAVs are highly immunogenic and have been the source of major public health successes in the fight against many viral diseases such as smallpox, measles, and rubella. However, LAVs have some drawbacks. The production of attenuated strains is usually based on empirical methods, their genetic stability is not always optimal, and their use in certain populations can lead to serious adverse events (44).

The first studies to investigate the phenotype of attenuated viruses by *in vivo* generation began in the 1990s and therefore focused on retroviruses. Work on caprine arthritis encephalitis virus (CAEV) made it possible to study the effect of *tat* gene deletion on viral phenotype (45, 46). Injecting the deleted but functional infectious clone induced protective but non-sterilising immunity in goats. In the 2000s, some attenuation work on viruses of veterinary interest, foot-and-mouth disease virus (FMDV) and porcine circovirus (PCV), was published, whereas the fight against arboviruses began to gain momentum (47, 48).

In 2003 and for the first time, a study involving a flavivirus reported the practical use of an LAV delivered in DNA form, known as a “DNA-launched LAV” (49). Hall et al. showed that injecting an infectious clone of the Kunjin strain of the WNV carrying an additional mutation in the NS1 protein was able to protect animals from infection by the wild-type NY99 strain. In 2016, Yamshikov et al. investigated the effect of several mutations from the SA14-14-2 strain of Japanese encephalitis virus (JEV) in an infectious WNV clone using DNA-launched infection in mice (50, 51). A recent study by Pushko’s team demonstrated the ability of plasmids containing the genome of the SA14-14-2 strain of JEV to stimulate an immune response in mice (52). This team and others have also shown that the 17D strain of yellow fever virus (YFV) could be delivered as DNA (53, 54). Moreover, one of these studies showed that this type of approach resulted in a decreased incidence of mutations in newly generated viral populations within mouse models, when compared to direct administration of the vaccine strain (55).

In the wake of the recent Zika virus (ZIKV) emergence, several DNA-launched LAV candidates had been in development. Research carried out by Pei-Yong Shi demonstrated that a DNA-launched LAV candidate induced immunogenicity in mice (56, 57). Recently, a study using a ZIKV vaccine chimera based on a genotype 2 dengue virus (DENV) backbone showed that inoculating viral particles or infectious DNA resulted in similar and protective immune responses (58).

Since 2012, DNA-launched LAVs had also been developed against alphaviruses. The work by Pushko’s team on Venezuelan equine encephalitis virus (VEEV) demonstrated the efficacy of DNA-launched LAVs encoding the TC83 and V4020 vaccine strains in inducing protective immunity in several animal models (59–61). This team obtained similar results by injecting an attenuated strain of chikungunya virus (CHIKV) (181/25 strain) in the form of cloned DNA into mice (62). DNA-launched LAVs possess the ideal vaccine profile as they result in potent long-term immunity and are convenient to produce, store, and transport. They also offer superior genetic stability when compared to conventional LAVs. Nonetheless, upstream attenuation work and vaccine strain development are crucial factors in LAVs design. Furthermore, additional research is required to validate the efficacy and safety of DNA-launched vaccines prior to their human application.

## OVERVIEW OF THE DIFFERENT STRATEGIES DEVELOPED

### Nature of the nucleic acid administered

Since the 1950s, a wide range of viruses have been generated *de novo* in animals using a variety of methods (Table 1).

**TABLE 1:** Nature of nucleic acids administrated to rescue virus *in vivo^a^*

Genome^*b*^	Family	Genre	Purified genome	*In vitro* RNA transcript	Cloned DNA
Linear ssRNA (+)	Arteriviridae	Betaarterivirus	–	PRRSV	–
		Gammaarterivirus	LDV	–	–
	Caliciviridae	Lagovirus	RHDV	RHDV	–
	Flaviviridae	Flavivirus	WNV, MVEV, TBEV, JEV, DENV, ITV	DENV	JEV, YFV, WNV, TBEV, ZIKV, DENV = ZIKV^*c*^
		Hepacivirus	–	GBV-B, HCV, GBV-B = HCV^*c*^	–
	Hepeviridae	Orthohepevirus	–	HEV	–
	Picornaviridae	Aphtovirus	FMDV	FMDV	FMDV
		Cardiovirus	EMCV, TMEV	–	–
		Enterovirus	PV	–	EV-A71
		Hepatovirus	–	HAV	–
	Togaviridae	Alphavirus	EEEV	–	CHIKV, VEEV
	Coronaviridae	Alphacoronavirus	α-CoV-1	–	–
	Retroviridae	Deltaretrovirus	BLV	–	HTLV, BLV
		Gammaretrovirus	–	–	FELV, MuLV
		Lentivirus	SIV	–	CAEV, SIV, FIV
Circular ssRNA (−)	Kolmioviridae	Deltavirus	–	–	HDV
Circular dbDNA	Papillomaviridae	Kappapapillomavirus	KPV2	–	–
	Polyomaviridae	–	PyV, SV40	–	PyV, MuPyV
Partially double-stranded circular DNA	Hepadnaviridae	Avihepadnavirus	–	–	DHBV
		Orthohepadnavirus	–	–	GSHV, HBV
Circular ssDNA genome (+)	Anelloviridae	Kappatorquevirus	–	–	TTSV
Circular ssDNA genome (−)	Circoviridae	Circovirus	–	–	PCV, DuCV
Linear dbDNA	Adenoviridae	Mastadenovirus	SAdV	–	–
	Herpesviridae	Rhadinovirus	AtGHV, SaGHV	–	–

^*a*^ Virus name abbreviations and further information can be found in the Supplemental material. '–' symbols mean that no virus has been found for these conditions.

^*b*^ '(+)' and '(−)' symbols means positive and negative genome polarities, respectively.

^*c*^ ‘=’ symbols between viral species represent chimeric virus (first species is the backbone).

Initially, the purified viral genome was used to inoculate animals. This method was gradually replaced by the use of reverse genetics systems.

Regarding RNA viruses, with the exception of hepatitis delta virus (HDV), all pathogens *de novo* generated are positive-stranded. Several viruses were rescued from synthetic RNA by producing RNA transcripts *in vitro*. The viral genome was cloned into a plasmid vector under the control of a eukaryotic SP6 or T7 promoter and then transcribed using molecular biology tools. The addition of a cap at the 5′ end was not essential to confer infectious potency, except in the case of HEV (43, 63).

When RNA viruses were produced following the administration of DNA, the infectious clones were plasmids or bacterial chromosomes, which were usually inoculated directly into animals or after a linearization step. The regulatory sequences in retroviral genomes alone were sufficient to generate this kind of virus directly *in vivo* (see Supplemental material). This was not the case for the other RNA viruses studied. The infectious clones of these other viruses consisted mainly of the viral genome flanked at the 5′ end by a eukaryotic promoter, such as the promoter of the cytomegalovirus (pCMV) or of the SV40 (pSV40). The addition of regulatory sequences at the 3′ end of the viral genome appeared to be optional, as the majority of viruses generated in their presence were also generated in their absence. This was particularly the case for YFV and the CHIKV (53, 54, 62, 64). The main terminator sequences used were the HDV ribozyme (HDVr), followed by the polyadenylation signal (Poly(a)) of SV40, or the bovine growth hormone transcriptional terminator.

In a recent work, several arboviruses have also been rescued by the administration of subgenomic DNA fragments (65). On the basis of the “Infectious Subgenomic Amplicons” method, DNA fragments, coding for the full-length genome flanked by transcription regulators, were amplified by polymerase chain reaction, equimolarly pooled, and administered to animals.

Several techniques were explored for DNA virus generation *in vivo*. Viruses with circular and linear double-stranded genomes were produced from purified genomes. Subsequently, viruses with double-stranded circular, partially double-stranded circular, and single-stranded genomes had been *de novo* generated by inoculation of cloned DNA, mainly hepadnaviruses and circoviruses. As their genome is naturally infectious, no regulatory sequence was required for their generation. However, some studies reported the use of regulatory sequences without providing a basis for comparison with a system that lacked them (9, 66, 67). For example, a type 2 PCV strain was generated in mice from a genome cloned into a bacterial plasmid under the control of the pCMV (66).

Reverse genetics was initially applied to DNA viruses, as they provided a relatively straightforward system for genetic manipulation. Regarding positive-strand RNA viruses, viral RNA synthesis can be achieved by generating an infectious clone of complementary DNA (cDNA) and integrating a RNA polymerase promoter, an approach that does not present significantly increased complexity (68). As a result, the majority of *de novo* generation of viruses *in vivo* efforts have focused on these specific viruses and represent the majority of scientific publications on the subject so far.

The reverse genetics of negative-strand and segmented RNA viruses necessitates significantly more complex tools. *In vitro* generation of these viruses is predominantly accomplished using cell lines that express recombinant proteins, such as viral polymerases, or through co-infection models involving helper viruses or plasmids. These approaches may present certain limitations for *in vivo* applications. To the best of our knowledge, the HDV is the only negative-strand RNA virus that has been successfully generated *de novo* to date. Notably, HDV replication is dependent on HBV infection, and its *de novo* generation *in vivo* was accomplished using chimpanzees with chronic HBV infection (69).

The reverse genetics of viruses carrying large genomes is also challenging and limits the study of these viruses using the aforementioned techniques. For instance, the genomes of viruses recovered *in vivo* using cloned DNA or artificial RNA transcripts did not exceed 16 kb (70). Conversely, there is only one example of DNA viruses of approximately 160 kb that has been recovered using purified genomes (71). Although current vectors allow the insertion of long sequences, such as YAC and BAC, they do not prevent the difficulty of cloning these sequences into the vectors, nor the emergence of viral genomic sequences toxic to the microorganisms used to amplify the cloned genomes.

### Animal models

**Fig 1:**
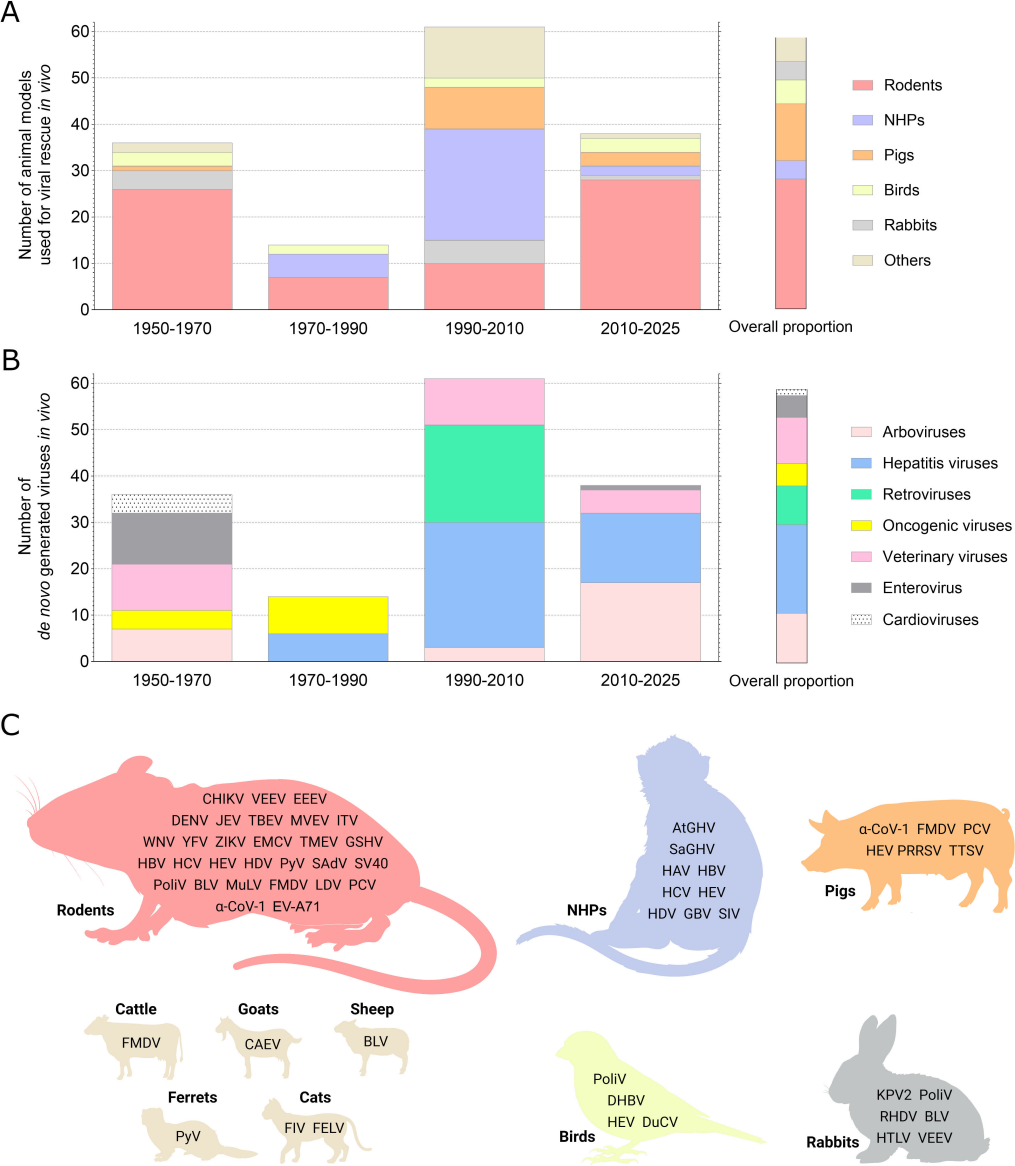
Overview of animal models used for *de novo* generation of viruses using nucleic acids. (**A**) Number of published animal models used for *de novo* generation of viruses *in vivo*. (**B**) Number of viruses *de novo* generated in published animal models. These charts show a total of 149 *de novo* generations of virus in 130 publications. (**C**) Viral species *de novo* generated in animal models. All rodent models are represented by the red mice shape (mice, hamsters, rats, cobaye, guinea pigs, beechey ground squirrels, and mongolian gerbils). NHPs means non-human primates. Virus name abbreviations and further information can be found in the Supplemental material.

Unsurprisingly, rodents have a prominent place in the arsenal of animal models used for *de novo* generation of viruses *in vivo* (Fig. 1).

Until the late 1980s, rodent models were predominant. The widespread adoption of the mouse model was attributed to the simplicity of viral isolation in their brain tissue. Additionally, the hamster and the guinea pig models were employed in various studies with FMDV and poliovirus during this period (72–75). The strong interest of the scientific community in hepatitis viruses has shifted the focus away from rodents toward non-human primate (NHP) models. In the 2000s, rodent models had been used to study flaviviruses and were adapted for the first time to *de novo* generate HBV and HCV viruses, which were previously exclusively studied in NHP models. There was a significant resurgence in their use since the early 2010s, particularly in research on hepatitis B and E and in the development of vaccine strategies for arboviruses. Among all studies utilizing rodents, some had used transgenic mouse models that are immunodeficient (43, 55, 58, 76–79) or humanized (expressing class I human leukocyte antigen) (54).

NHP models had become increasingly popular since the late 1970s, reaching their pinnacle between 1990 and 2010. Initially, they played a crucial role in the research of viral hepatitis because of their resemblance to humans. However, rodent models with fewer constraints were more frequently used for HBV studies since the 2000s, while avian and porcine models were preferred for HEV research. While most studies concerning the *de novo* generation of retroviruses focused on viruses that infect other animal models, the recent investigations employed DNA infection models with the SIV genome in macaque monkeys since the early 2000s (15–17). Most NHPs used for *de novo* generation of viruses belong to the *Catarrhines* suborder (Old World), which comprises various species of macaques and chimpanzees. Nevertheless, some NHPs from the *Platyrhinian* suborder (New World) were also used, notably for the first *de novo* generation using NHPs with tamarin-specific herpesviruses (71).

Since the 1980s, pigs, chickens, and ducks had been predominantly used for the *de novo* generation and study of hepatitis viruses, which are particularly relevant for these models. In fact, some viruses, such as porcine and avian circoviruses, became important in veterinary healthcare due to the increase in intensive farming practices (47, 48, 66, 67, 80).

In the early investigations, rabbits, like birds, were used to establish the pathogenicity potential of viral genomes (3, 73, 75, 81). Rabbits have been used for research on retroviruses and rabbit hemorrhagic disease virus (RHDV), a virus of veterinary interest (22, 23, 82, 83). In 2022, the rabbit model was employed to assess the immunogenicity of a DNA-launched LAV against the VEEV (61).

Other animal models, such as cats, sheep, goats, and cattle, had been used to *de novo* generate retroviruses like FIV and BLV. We also report the use of the ferret model, although only in a single study in 1961 to demonstrate the oncogenic potential of a polyomavirus genome (8).

The age of the animals is a crucial factor from an immunological perspective. Neonatal or juvenile animals were preferred to facilitate *de novo* virus generation. Nevertheless, no comparative studies had been found between animals of varying ages. In the absence of knowledge about the impact of animal age on the *de novo* generation of viruses *in vivo*, parameters already applied in classical infectious models should be followed to, at least, facilitate the replication of the virus once generated.

### Inoculation routes

As with animal models, the inoculation route is typically tailored to fit the virus’s tropism being studied. However, certain routes offer advantages in terms of nucleic acid transfection or immune response induction.

Many viruses had been *de novo* generated in animals by directly targeting the tissues for which the virus of interest has a high natural tropism (e.g., the intrahepatic route widely used for hepatitis viruses). In addition, other specific routes had been used, such as intra-articular injection for CAEV, intralymphoid route for PCV, and intralingual injection for FMDV (45, 46, 48, 84). In the early studies, intracerebral injection was extensively employed as the brain tissue facilitated the viral replication and the isolation of numerous time-consuming viruses.

Nucleic acids were commonly delivered systemically via intraperitoneal (IP), IV, or SC routes. For instance, YFV and ZIKV flaviviruses were generated through the IP and the SC routes, whereas HCV was generated through the IV route (55, 76, 78). However, these routes have the disadvantage of diluting nucleic acids in a large volume of tissue, which limits transfection efficiency. In spite of this, a recent study examining multiple methods for *in vivo* ZIKV DNA generation achieved superior outcomes with the IP route compared to the SC or the IM routes (76).

Unlike systemic administration, the ID route allows nucleic acids to be sequestered in a confined area, promoting cellular penetration at the injection site. During the 1990s, this technique was used to generate numerous retroviral wild-type strains, including BLV (22). Using the same virus and the same route, first attenuated strains were *de novo* generated in animals (25, 85). This route is widely used in immunization procedures because the skin contains large numbers of immune sentinel cells, rapidly delivering antigens to lymphoid organs and thereby facilitating the development of adaptive immunity. Furthermore, when combined with some transfection strategies such as electroporation and biolistic inoculation, this technique allowed the generation of attenuated strains of CHIKV and WNV in animals (17, 50, 64).

IM injections were commonly conducted into the striated skeletal muscle, although it is not a natural route of infection and viruses studied were not considered to have a tropism for the muscular tissue. Nevertheless, this route offers several advantages for transfection, as it provides access to a highly vascularized and easily accessible tissue composed of syncytial cells, especially myocytes, and allowing long-term expression of transgenes (86, 87). Similar to the ID route, it was used in the 1990s to produce many retroviruses such as SIV and HTLV (13, 82). Since then, it was used in combination with electroporation to produce vaccine strains for several flaviviruses, including YFV and ZIKV (53, 77).

### Formulation and physical delivery processes

**Fig 2:**
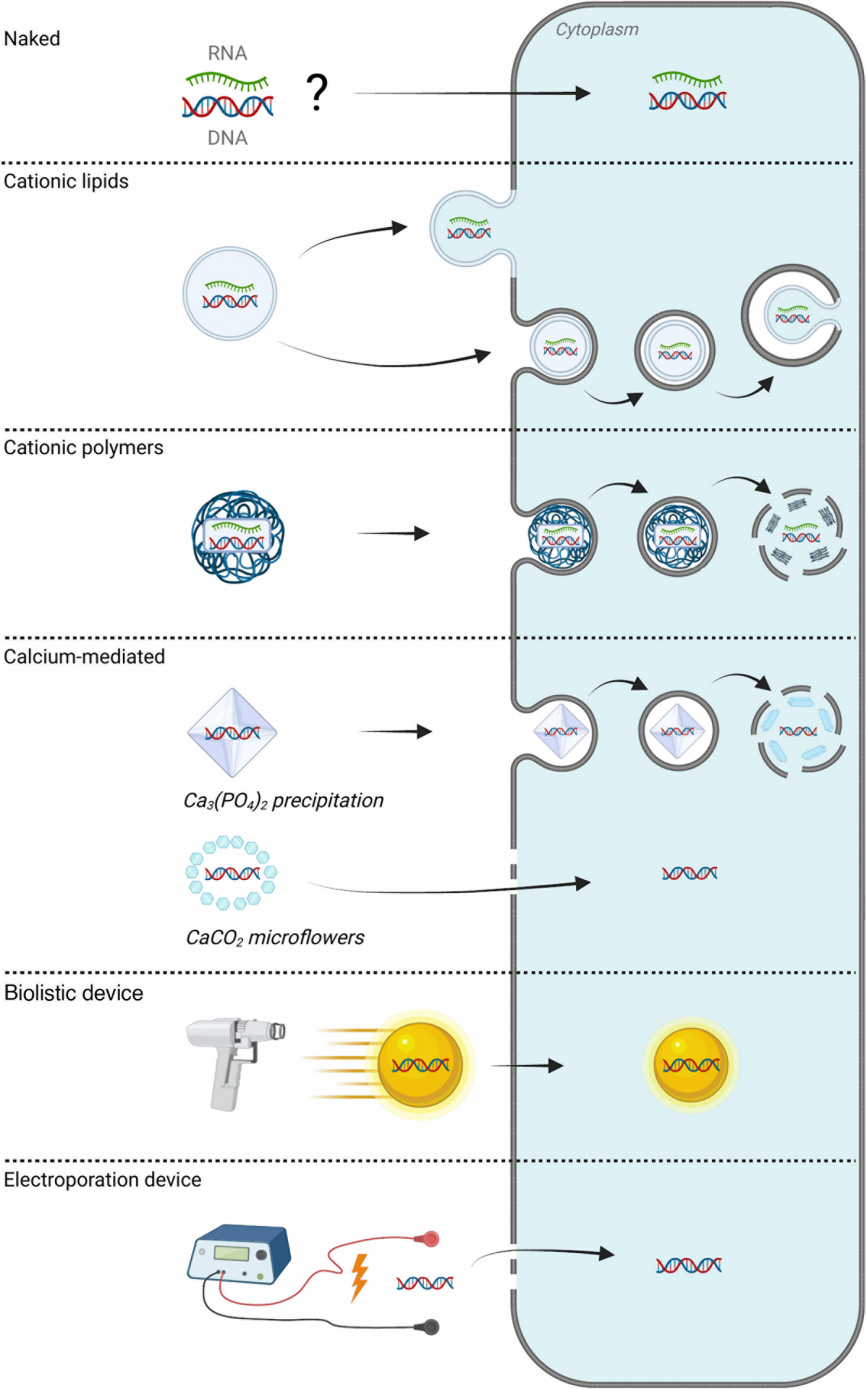
Summary of nucleic acid transfection methods and penetration mechanisms used for *de novo* generation of viruses *in vivo*. Further information can be found in the Supplemental material.

Viruses have been generated *de novo* in animals using many transfection protocols (Fig. 2). Although some comparative studies have examined different *de novo* generation methods, the summary of relevant information concerning the doses and transfection systems used *in vivo* is quite challenging. Indeed, most of the studies aimed to achieve maximum efficiency and therefore inoculate large quantities of nucleic acid. In addition to the injection method and nucleic acid formulation, the efficiency of *de novo* generation of viruses also depends on the nature of the genetic material and the animal model used.

### Chemical formulations

#### Naked DNA

Injection of a simple aqueous saline solution (often NaCl or PBS) containing naked nucleic acids, without any specific formulation or inoculation method, has been widely used and effective for most viruses *de novo* generated in animal models (see Supplemental material). However, the mechanism of cell uptake of free nucleic acids from the extracellular matrix is not fully understood. A number of hypotheses have been proposed. These include physical disruption of the cell membrane by injection with a needle, the appearance of spontaneous pores or pores induced by the pressure exerted on the cells during injection, or active capture by endocytic pathways (88, 89). For example, a study demonstrated the role of macropinocytosis in the uptake of naked DNA by mesothelial cells in the stomach of mice (90).

#### Cationic lipids

Cationic lipids are very common transfection agents. The formation of liposomes in the presence of nucleic acid creates a protective lipid bilayer around the nucleic acid. On contact with cells, the nucleic acid is released into the cytoplasm by fusion with the endosomal membrane following endocytosis of the lipid-nucleic acid complex, also named lipoplex. Less frequently, the lipoplex may fuse directly with the cell membrane from the extracellular matrix (lipofection) (91, 92). Lipoplexes were used to *de novo* generate several RNA and DNA viruses *in vivo*, using commercial or home-made lipid preparations. Work on ZIKV compared generation by injection of naked DNA or lipoplexes: naked DNA doses of 50–100 µg were required to cause infection in 100% of the transfected animals, while lipoplexed DNA gave equivalent results at doses of 6–13 µg (76).

#### Cationic polymers

Cationic polymers are well-known transfection agents that condense nucleic acids by electrostatic binding and adhere to the cell membrane (93). As with lipoplexes, polymers have been used to *de novo* generate viruses *in vivo* using commercial or home-made preparations. The most common polymer was diethylaminoethyl-dextran (DEAE-Dextran), a polysaccharide with a weak potency but a low level of toxicity (91). In particular, it facilitated the generation of viruses in animals from purified genomes of DNA and RNA viruses (23, 83). We also highlighted the recent use of a commercial preparation combining the use of the chitosan polymer and cationic lipids to generate ZIKV by IP inoculation of an infectious clone (76).

#### Calcium-mediated transfection

Calcium-mediated transfection methods are simple and inexpensive approaches for delivering nucleic acids. However, they had only been used to *de novo* generate viruses *in vivo* a few times by inoculating DNA (55, 71, 94–96). Precipitating nucleic acids with calcium phosphate ((Ca_3_(PO_4_)_2_) is more popular and is the oldest transfection method described (97). It has the advantage of using a biocompatible and degradable substrate (91, 98). Ca_3_(PO_4_)_2_ can bind and protect nucleic acids as they are taken by endocytosis. It appears that the dissolution of calcium phosphate allows nucleic acids to escape from the endosome (99). As an example, this method was used to generate ground squirrel hepatitis virus in its natural host via the intrahepatic route (94). Calcium carbonate (CaCO_2_) microflowers represent another calcium-mediated transfection method that has not been widely used. It is thought that CaCO_2_ microflowers induce the formation of micropores on the surface of tissues, allowing better uptake of nucleic acids (100). This method had recently been used to generate YFV-17D vaccine strain by IP inoculation in mice (55).

### Physical delivery tools

Physical methods of nucleic acid delivery had also been developed to promote transfection. We only reported their uses to enhance *de novo* generation of viruses using DNA injection.

#### Biolistic injection

The Gene Gun biolistic technique is a needle-free injection method. This involves the injection of nucleic acids, adsorbed on gold or tungsten particles, onto the surface of a specific tissue (93, 101). The high pressure exerted during the injection process allows a proportion of the particles to penetrate directly into the cytoplasm of the cells. Few studies had been conducted using this method for the *de novo* generation of virus *in vivo*, but it had always been used as a tool for ID injection. Work on WNV has compared inoculation of attenuated strains by the conventional IM route or using the Gene Gun on the skin of mice (50). Gene gun ID inoculation resulted in superior immune response induction using 1 µg or 100 ng of DNA. However, it also led to increased mortality in mice.

#### Electroporation

Developed to enhance gene transfer, electroporation technically consists of the application of an electric field to a target tissue (93, 101). This induces cell membrane permeabilization and facilitates transfection with pre-inoculated nucleic acids. Furthermore, electroporation elicits immune cell recruitment by its pro-inflammatory effect, which is beneficial in the context of vaccination (102). For example, the inoculation of NHPs using a DNA vaccine encoding the HIV gag gene showed that IM electroporation, despite using 20 times less nucleic acid, generated a more potent immune response than conventional IM inoculation (103). For *de novo* generation of viruses, electroporation was mainly coupled with IM inoculation for many arboviruses such as tick-borne encephalitis virus (TBEV), JEV, YFV, ZIKV, and CHIKV. A study showed that IM electroporation of 0.01 µg of DNA encoding an attenuated strain of ZIKV induced viremia and humoral immunity comparable to a simple IM inoculation of 1 µg (77).

#### Hydrodynamic injection

Hydrodynamic injection is a technique that allows systemic inoculation by rapidly delivering, through the tail vein, a large volume of fluid carrying the nucleic acid of interest (104). Hepatocytes were the main target of this technique (105). For the *de novo* generation of viruses *in vivo*, only the work led by Tang on HBV mentioned its use, which resulted in a transient infection of the animals (106–111). However, hydrodynamic injection presents risks for the injected recipients, notably major cardiovascular complications already observed in animals (112).

## CONCLUSION

This review provides a comprehensive analysis of studies utilizing nucleic acid-based infectious material *in vivo*. For over six decades, the *de novo* generation of viruses in animal hosts has been employed for a variety of research purposes, relying on a wide range of evolving methodologies. This approach continues to play a significant role in virological research, as it enables the investigation of complex virus–host interactions within a physiologically relevant context—an essential prerequisite for a detailed understanding of pathogenic mechanisms, viral replication dynamics, and associated immune responses. Although this approach has greatly benefited from recent advancements in transfection techniques, experimental methodologies, and knowledge gained during the COVID-19 pandemic—particularly those related to mRNA vaccine technologies—current reverse genetics methods used for *in vivo de novo* virus generation still face several limitations. These are especially pronounced when dealing with segmented genome viruses, negative-sense single-stranded RNA viruses, or viruses with genomes exceeding 15 kb.

Originally employed to simplify the study of biological and molecular properties of viruses, the subsequent *de novo* generation of viruses in animal hosts emerged as a valuable tool for exploring alternative strategies of vaccination. Nucleic acid-based LAVs, particularly those based on DNA, represent a promising category of protective strategies, offering many of the same advantages as traditional LAVs. They are capable of eliciting robust and long-lasting immune responses, while also presenting logistical advantages such as enhanced stability, low production costs, and ease of distribution. Importantly, they have the potential to induce both humoral (B cell-mediated) and cellular (T cell-mediated) immunity. Additionally, factors such as the transfection method, injection site, and use of adjuvants can significantly influence the quality and magnitude of the immune response, including the activation of CD4^+^ and CD8^+^ T lymphocytes as well as macrophages (113).

The application of this technology nevertheless raises complex questions. First, most of these concepts have been studied in rodents, and their extrapolation to higher species, including humans, requires in-depth evaluation, particularly with regard to immune mechanisms. Moreover, as previously described for classical LAVs (114, 115) and more recently emphasized for self-amplifying mRNA vaccines (116), concerns have been raised regarding the potential for chromosomal integration into the host genome, which warrants further investigation.

Research and development in nucleic acid-based LAVs is a rapidly advancing field. While no clinical trials involving these strategies have yet been initiated in humans, several recent studies on DNA-based LAVs have received funding from government institutions such as the NIH and NIAID, highlighting the increasing interest in this approach and the scientific community’s commitment to gaining a deeper understanding of these techniques.

## SUPPLEMENTAL MATERIAL

Supplemental material. msphere.00742-24-s0001.pdf Publications of *de novo* generation of virus *in vivo*.

ASM does not own the copyrights to Supplemental Material that may be linked to, or accessed through, an article. The authors have granted ASM a non-exclusive, world-wide license to publish the Supplemental Material files. Please contact the corresponding author directly for reuse.
