# Supplementary material for: De novo generation of viruses in animals: from infection models to vaccine development
Source: mSphere. 2025 Aug 11;10(9):e00742-24. doi: 10.1128/msphere.00742-24 (PMC12482153; doi:10.1128/msphere.00742-24)
Supplement: Supplemental material. — Publications of de novo generation of virus in vivo. [file msphere.00742-24-s0001.pdf]

Supplementary Data : Publications of *de novo* generation of virus *in vivo*. Reference numbers from this table do not match with those of the main manuscript. A list of abbreviation is presented below the table.

Article		Virus			Nucleic acid injected (Table2)			Regulatory sequences		Animal model			Transfection		
**Year**	**Ref.**	**Genus**	**Species**	**Category (Fig.1)**	**Matrix**	**Genome**	**Vector**	**5' end**	**3' end**	**Animal**	**Category (Fig.2)**	**Specificity**	**Route**	**Method**	**Formulation**
1957	[1]	*Cardiovirus*	EMCV	Cardiovirus	RNA	Purified	-	-	-	Mice	Rodent	-	IC	Naked	-
	[2]	*Flavivirus*	WNV	Arbovirus	RNA	Purified	-	-	-	Mice	Rodent	-	IC	Naked	-
		Enterovirus	Poliovirus	Enterovirus		Purified	-	-	-	Mice	Rodent	-	IC	Naked	-
	[3]	*Alphavirus*	EEEV	Arbovirus	RNA	Purified	-	-	-	Mice	Rodent	-	IC	Naked	-
1958	[4]	*Aphtovirus*	FMDV	Veterinary virus	RNA	Purified	-	-	-	Mice	Rodent	-	IM	Naked	-
1959	[5]	*Flavivirus*	MVEV	Arbovirus	RNA	Purified	-	-	-	Mice	Rodent	-	IC	Naked	-
	[6]	*Cardiovirus*	TMEV	Cardiovirus	RNA	Purified	-	-	-	Mice	Rodent	-	IC	Naked	-
	[7]	*Enterovirus*	Poliovirus	Enterovirus	RNA	Purified	-	-	-	Chicken	Bird	-	IC	Naked	-
										Rabbit	Rabbit	-	IC	Naked	-
										Cobaye	Rodent	-	IC	Naked	-
										Hamster	Rodent	-	IC	Naked	-
	[8]	*Enterovirus*	Poliovirus	Enterovirus	RNA	Purified	-	-	-	Chicken	Bird	-	IC	Naked	-
										Rabbit	Rabbit	-	IC	Naked	-
										Cobaye	Rodent	-	IC	Naked	-
										Hamster	Rodent	-	IC	Naked	-
										Mice	Rodent	-	IC	Naked	-
	[9]	*Flavivirus*	TBEV	Arbovirus	RNA	Purified	-	-	-	Mice	Rodent	-	IC	Naked	-
	[10]	*Aphtovirus*	FMDV	Veterinary virus	RNA	Purified	-	-	-	Cow	Other	-	NR	Naked	-
										Cobaye	Rodent	-	NR	Naked	-
	[11]	*Aphtovirus*	FMDV	Veterinary virus	RNA	Purified	-	-	-	Cobaye	Rodent	-	ID	Naked	-
										Mice	Rodent	-	ID	Naked	-
1960	[12]	*Kappapapillomavirus*	KPV 2	Oncogenic virus	DNA	Purified	-	-	-	Rabbit	Rabbit	-	ID	Naked	-
1961	[13]	*Polyomavirus*	PyV	Oncogenic virus	DNA	Purified	-	-	-	Furret	Other	-	SC	Naked	-
	[14]	*Kappapapillomavirus*	KPV 2	Oncogenic virus	DNA	Purified	-	-	-	Rabbit	Rabbit	-	ID	Naked	-
	[15]	*Flavivirus*	DENV	Arbovirus	RNA	Purified	-	-	-	Mice	Rodent	-	IC	Naked	-
			JEV	Arbovirus	RNA	Purified	-	-	-	Mice	Rodent	-	IC	Naked	-
1963	[16]	*Gammaarterivirus*	LDV	Veterinary virus	RNA	Purified	-	-	-	Mice	Rodent	-	IC	Naked	-
1964	[17]	*Gammaarterivirus*	LDV	Veterinary virus	RNA	Purified	-	-	-	Mice	Rodent	-	IC	Naked	-
	[18]	*Polyomavirus*	PyV	Oncogenic virus	DNA	Purified	-	-	-	Hamster	Rodent	-	SC or IC	Naked	-
1965	[19]	*Gammaarterivirus*	LDV	Veterinary virus	RNA	Purified	-	-	-	Mice	Rodent	-	IM or IC	Naked	-
	[20]	*Enterovirus*	Poliovirus	Enterovirus	RNA	Purified	-	-	-	Chicken	Bird	-	IC	Naked	-

1966	[21]	*Cardiovirus*	EMCV	Cardiovirus	RNA	Purified	-	-	-	Mice	Rodent	-	Oronasal	Naked	-
1968	[22]	*Cardiovirus*	EMCV	Cardiovirus	RNA	Purified	-	-	-	Mice	Rodent	-	Oronasal	Cationic polymers	DEAE-dextran
	[23]	*Mastadenovirus*	SAdV	Oncogenic virus	DNA	Purified	-	-	-	Hamster	Rodent	-	SC	Naked	-
	[24]	*Alphacaronavirus*	α-CoV-1	Veterinary virus	RNA	Purified	-	-	-	Pig	Pig	-	Oronasal	Naked or Cationic polymers	DEAE-dextran
										Mice	Rodent	-	IC	Naked or Cationic polymers	DEAE-dextran
1970	[25]	*Flavivirus*	ITV	Arbovirus	RNA	Purified	-	-	-	Mice	Rodent	-	IC	Naked	-
1971	[26]	*Adenovirus*	SAdV	Oncogenic virus	DNA	Purified	-	-	-	Hamster	Rodent	-	SC	Naked	-
1977	[27]	*Polyomavirus*	SV40	Oncogenic virus	DNA	Purified	-	-	-	Hamster	Rodent	-	SC	Naked	-
1978	[28]	*Rhadinovirus*	AtGHV	Oncogenic virus	DNA	Purified	-	-	-	Saginus oedipus	NHP	-	IV, IP, IM combined	Calcium meditated	Calcium phosphate
			SaGHV	Oncogenic virus	DNA	Purified	-	-	-	Saginus oedipus	NHP	-	IV, IP, IM combined	Calcium meditated	Calcium phosphate
1979	[29]	*Polyomavirus*	PyV	Oncogenic virus	DNA	Purified	-	-	-	Hamster	Rodent	-	IP	Naked	-
										Mice	Rodent	-	SC or IP or Oral or Rectal	Naked	-
1982	[30]	*Orthohepadnavirus*	HBV	Hepatitis virus	DNA	Cloned	Plasmid or excised genome	pSV40	-	Pan troglodytes	NHP	-	Intrahepatic + IV + IM	Cationic polymers	DEAE-dextran
1984	[31]	*Polyomavirus*	MuPyV	Oncogenic virus	DNA	Cloned	Plasmid	-	-	Mice	Rodent	-	Intrahepatic or intrasplenic	Calcium meditated	Calcium phosphate
	[32]	*Orthohepadnavirus*	GSHV	Hepatitis virus	DNA	Cloned	Plasmid	-	-	Beechey ground squirrel	Rodent	-	Intrahepatic	Naked or Calcium mediated	Calcium phosphate
	[33]	*Avihepadnavirus*	DHBV	Hepatitis virus	DNA	Cloned	Plasmid or excised genome	-	-	Duck	Bird	-	Intrahepatic	Naked	-
1985	[34]	*Orthohepadnavirus*	HBV	Hepatitis virus	DNA	Cloned	Excised genome	-	-	Pan troglodytes	NHP	-	Intrahepatic	Naked or Cationic polymers	DEAE-dextran
1987	[35]	*Avihepadnavirus*	DHBV	Hepatitis virus	DNA	Cloned	Plasmid or excised genome	-	-	Duck	Bird	-	Intrahepatic	Cationic polymers	DEAE-dextran
1989	[36]	*Deltavirus*	HDV	Hepatitis virus	DNA	Cloned	Plasmid	pSV40	SV40-poly(A)	Pan troglodytes	NHP	-	Intrahepatic	Cationic polymers	DEAE-dextran
1990	[37]	*Lagovirus*	RHDV	Veterinary virus	RNA	Purified	-	-	-	Rabbit	Rabbit	-	Intrahepatic	Cationic polymers	DEAE-dextran
1991	[38]	*Lentivirus*	SIV	Retrovirus	DNA	Cloned	Plasmid	-	-	Macaca fascicularis	NHP	-	IM	Naked	-
1992	[39]	*Hepatovirus*	HAV	Hepatitis virus	RNA	Transcript	Uncapped RNA	pSP6	-	Ouistiti	NHP	-	Intrahepatic	Naked	-
	[40]	*Gammaretrovirus*	MuLV	Retrovirus	DNA	Cloned	Plasmid or excised genome	-	-	Mice	Rodent	-	IP	Naked	-

1992	[41]	*Deltarétrovirus*	BLV	Retrovirus	DNA	Cloned	Plasmid	-	-	Sheep	Other	-	ID	Naked or Cationic polymers or lipolexe	DEAE-dextran or DOTAP
										Rabbit	Rabbit	-	ID	Naked or Cationic polymers or lipolexe	DEAE-dextran or DOTAP
1993	[42]	*Deltarétrovirus*	BLV	Retrovirus	DNA	Cloned	Plasmid	-	-	Sheep	Other	-	ID	Cationic lipids	DOTAP
1994	[43]	*Deltarétrovirus*	BLV	Retrovirus	DNA	Cloned	Plasmid	-	-	Sheep	Other	-	ID	Cationic lipids	DOTAP
1995	[44]	*Lentivirus*	CAEV	Retrovirus	DNA	Cloned	Plasmid	-	-	Goat	Other	-	Intraarticular	Cationic lipids	DOTAP
	[45]	*Deltavirus*	HDV	Hepatitis virus	DNA	Cloned	Plasmid	pSV40 or pCMV	-	Mice	Rodent	-	IM	Naked	-
	[46]	*Hepatovirus*	HAV	Hepatitis virus	RNA	Transcript	Uncapped RNA	pSP6	-	Saginus Mystax	NHP	-	Intrahepatic	Naked	-
1996	[47]	*Deltarétrovirus*	HTLV	Retrovirus	DNA	Cloned	Plasmid	-	-	Rabbit	Rabbit	-	IM	Naked	-
1997	[48]	*Deltarétrovirus*	BLV	Retrovirus	DNA	Cloned	Plasmid	No or SNV transcriptional sequences	No or SNV transcriptional sequences	Rat	Rodent	-	SC	Cationic polymers	Dimethylsulfoxide polybrene
	[49]	*Hepacivirus*	HCV	Hepatitis virus	RNA	Transcript	Uncapped RNA	pT7	-	Pan troglodytes	NHP	-	Intrahepatic	Naked	-
	[50]	*Lentivirus*	FIV	Retrovirus	DNA	Cloned	Plasmid	-	-	Cat	Other	-	IM	Naked	-
	[51]	*Lentivirus*	FIV	Retrovirus	DNA	Cloned	Plasmid	SV40 - Tata box	-	Cat	Other	-	IM or ID	Naked	-
	[52]	*Aphtovirus*	FMDV	Veterinary virus	RNA	Transcript	Uncapped RNA	pT7		Mice	Rodent	-	IM or ID	Naked	-
	[53]	*Hepacivirus*	HCV	Hepatitis virus	RNA	Transcript	Uncapped RNA	pT7	-	Pan troglodytes	NHP	-	Intrahepatic	Naked	-
1998	[54]	*Gammaretrovirus*	FeLV	Retrovirus	DNA	Cloned	Plasmid	-	-	Cat	Other	-	ID	Cationic lipids	DOTAP
	[55]	*Lentivirus*	CAEV	Retrovirus	DNA	Cloned	Plasmid	-	-	Goat	Other	-	Intraarticular	Cationic lipids	DOTAP
1999	[56]	*Aphtovirus*	FMDV	Veterinary virus	DNA	Cloned	Plasmid	pCMV	HDVr - BGHTT	Pig	Pig	-	ID (Gene Gun)	Gene Gun	Gold nanoparticles
	[57]	*Hepacivirus*	HCV	Hepatitis virus	RNA	Transcript	Uncapped RNA	pT7	HDVr	Pan troglodytes	NHP	-	Intrahepatic	Naked	-
	[58]	*Hepacivirus*	GBV-B	Hepatitis virus	RNA	Transcript	Uncapped RNA	pT7	-	Saginus Mystax	NHP	-	Intrahepatic	Naked	-
	[59]	*Hepacivirus*	HCV	Hepatitis virus	RNA	Transcript	Uncapped RNA	pT7	-	Pan troglodytes	NHP	-	Intrahepatic	Naked	-
	[60]	*Deltarétrovirus*	BLV	Retrovirus	DNA	Cloned	Plasmid	No or SNV transcriptional sequences	No or SNV transcriptional sequences	Rabbit	Rabbit	-	ID	Cationic polymers	DEAE-dextran
	[61]	*Lentivirus*	SIV	Retrovirus	DNA	Cloned	Plasmid	-	-	Rhesus monkey	NHP	-	IM	Naked	-
	[62]	*Hepacivirus*	HCV	Hepatitis virus	RNA	Transcript	Uncapped RNA	pT7	-	Pan troglodytes	NHP	-	Intrahepatic	Naked	-
	[63]	*Hepacivirus*	HCV	Hepatitis virus	RNA	Transcript	Uncapped RNA	pT7	-	Pan troglodytes	NHP	-	Intrahepatic	Naked	-
2000	[64]	*Hepacivirus*	HCV	Hepatitis virus	RNA	Transcript	Uncapped RNA	pT7	-	Pan troglodytes	NHP	-	Intrahepatic	Naked	-

2000	[65]	*Hepacivirus*	HCV	Hepatitis virus	RNA	Transcript	Uncapped RNA	pT7	-	Pan troglodytes	NHP	-	Intrahepatic	Naked or Cationic lipids	"Lipofectin" kit
	[66]	*Lentivirus*	FIV	Retrovirus	DNA	Cloned	Plasmid	-	-	Cat	Other	-	ID	Cationic lipids	DOTAP
2001	[67]	*Orthohepevirus*	HEV	Hepatitis virus	RNA	Transcript	Capped or uncapped RNA	pT7	-	Pan troglodytes	NHP	-	Intrahepatic	Naked	-
										Rhesus monkey	NHP	-	Intrahepatic	Naked	-
	[68]	*Lentivirus*	SIV	Retrovirus	DNA	Cloned	Plasmid	-	-	Macaca nemestrina	NHP	-	IM or ID (Genegun)	Naked or Gene Gun	NR
	[69]	*Hepacivirus*	HCV	Hepatitis virus	RNA	Transcript	Uncapped RNA	pT7	-	Pan troglodytes	NHP	-	Intrahepatic	NR (supposed naked)	-
	[70]	*Deltarétrovirus*	BLV	Retrovirus	DNA	Cloned	Plasmid	-	-	Sheep	Other	-	ID	Cationic lipids	DOTAP
	[71]	*Lentivirus*	SIV	Retrovirus	DNA	Cloned	Plasmid	-	-	Rhesus monkey	NHP	-	IM	Naked	-
	[72]	*Deltarétrovirus*	BLV	Retrovirus	DNA	Cloned	Plasmid	-	-	Sheep	Other	-	ID	Cationic lipids	DOTAP
2002	[73]	*Hepacivirus*	HCV	Hepatitis virus	RNA	Transcript	Uncapped RNA	pT7	-	Pan troglodytes	NHP	-	Intrahepatic	Naked	-
	[74]	*Circovirus*	PCV	Veterinary virus	DNA	Cloned	Plasmid	-	-	Pig	Pig	-	Intrahepatic or intralymphoïdal	Naked	-
2003	[75]	*Aphtovirus*	FMDV	Veterinary virus	RNA	Transcript	Uncapped RNA	pSP6	-	Mice	Rodent	-	IP	Cationic lipids	"Lipofectamine 2000" kit
	[76]	*Circovirus*	PCV	Veterinary virus	DNA	Cloned	Plasmid	-	-	Pig	Pig	-	Intralymphoïdal	Naked	-
	[77]	*Flavivirus*	WNV	Arbovirus	DNA	Cloned	Plasmid	pCMV	HDVr	Mice	Rodent	-	IM	Naked	-
	[78]	*Lentivirus*	SIV	Retrovirus	DNA	Cloned	Plasmid	-	-	Rhesus monkey	NHP	-	IM	Naked	-
	[79]	*Hepacivirus*	HCV	Hepatitis virus	RNA	Transcript	Uncapped RNA	pT7	-	Pan troglodytes	NHP	-	Intrahepatic	Naked	-
2004	[80]	*Hepacivirus*	HCV	Hepatitis virus	RNA	Transcript	Uncapped RNA	pT7	-	Transgenic mice	Rodent	NOD/SCID	IV	Naked	-
	[81]	*Hepacivirus*	GBV-B	Hepatitis virus	RNA	Transcript	Uncapped RNA	pT7	-	Saginus Mystax	NHP	-	Intrahepatic	Naked	-
	[82]	*Circovirus*	PCV	Veterinary virus	DNA	Cloned	Plasmid	-	-	Pig	Pig	-	IM or IP	Naked	-
2005	[83]	*Orthohepevirus*	HEV	Hepatitis virus	RNA	Transcript	Capped RNA	pT7		Chicken	Bird	-	Intrahepatic	Naked	-
	[84]	*Orthohepevirus*	HEV	Hepatitis virus	RNA	Transcript	Capped RNA	pT7	-	Pig	Pig	-	Intrahepatic	Naked	-
	[85]	*Flavivirus*	DENV	Arbovirus	RNA	Transcript	Capped RNA	pSP6		Mice	Rodent	-	IC	Naked	-
2006	[86]	*Lagovirus*	RHDV	Veterinary virus	RNA	Transcript	Uncapped RNA	pSP6		Rabbit	Rabbit	-	IP or Intrahepatic	Cationic lipids	"Lipofectin" kit
	[87]	*Flavivirus*	WNV	Arbovirus	DNA	Cloned	Plasmid	pCMV	HDVr - BGHTT	Mice	Rodent	-	IM	Naked	-
2007	[88]	*Hepacivirus*	GBV-B	Hepatitis virus	RNA	Transcript	Uncapped RNA	pT7	-	Ouistiti commun	NHP	-	Intrahepatic	Naked	-
	[89]	*Orthohepevirus*	HEV	Hepatitis virus	RNA	Transcript	Capped RNA	pT7		Pig	Pig	-	Intrahepatic	Naked	-
	[90]	*Betaarterivirus*	PRRSV	Veterinary virus	RNA	Transcript	Capped RNA	pT7		Pig	Pig	-	Intralymphoïdal	Naked	-

2007	[91]	*Orthohepadnavirus*	HBV	Hepatitis virus	DNA	Cloned	Plasmid	-	-	Mice	Rodent	-	IV (hydrodynamic)	Hydrodynamic	-
	[92]	*Hepacivirus*	HCV	Hepatitis virus	RNA	Transcript	Uncapped RNA	pT7	-	Pan troglodytes	NHP	-	Intrahepatic	Naked	-
2009	[93]	*Orthohepevirus*	HEV	Hepatitis virus	RNA	Transcript	Capped RNA	pT7		Chicken	Bird	-	Intrahepatic	Naked	-
										Pig	Pig	-	Intrahepatic	Naked	-
	[94]	*Aphtovirus*	FMDV	Veterinary virus	RNA	Transcript	Uncapped RNA	pSP6		Pig	Pig	-	ID	Cationic lipids	"Lipofectin" kit
2010	[95]	*Hepacivirus*	HCV	Hepatitis virus	RNA	Transcript	Uncapped RNA	pT7	-	Pan troglodytes	NHP	-	Intrahepatic	Naked	-
2011	[96]	*Orthohepevirus*	HEV	Hepatitis virus	RNA	Transcript	Capped RNA	pT7	-	Pig	Pig	-	Intrahepatic	Naked	-
	[97]	*Orthohepevirus*	HEV	Hepatitis virus	RNA	Transcript	Capped RNA	pT7		Chicken	Bird	-	Intrahepatic	Naked	-
2012	[98]	*Orthohepevirus*	HEV	Hepatitis virus	RNA	Transcript	Capped RNA	pT7		Pig	Pig	-	Intrahepatic	Naked	-
	[99]	*Orthohepadnavirus*	HBV	Hepatitis virus	DNA	Cloned	Plasmid	-	-	Mice	Rodent	-	IV (hydrodynamic)	Hydrodynamic	-
	[100]	*Annelovirus*	TTSV	Veterinary virus	DNA	Cloned	Plasmid	-	-	Pig	Pig	-	Intralymphoïdal + IM	Naked	-
	[101]	*Orthohepadnavirus*	HBV	Hepatitis virus	DNA	Cloned	Plasmid	-	-	Mice	Rodent	-	IV (hydrodynamic)	Hydrodynamic	-
2013	[102]	*Alphavirus*	VEEV	Arbovirus	DNA	Cloned	Plasmid	pCMV	-	Mice	Rodent	-	IM + EP	EP	-
	[103]	*Orthohepevirus*	HEV	Hepatitis virus	RNA	Transcript	Capped RNA	pT7		Rat	Rodent	-	Intrahepatic	Naked	-
2014	[104]	*Alphavirus*	CHIKV	Arbovirus	DNA	Cloned	Alphavirus DNA replicon (DREP)	pCMV	-	Mice	Rodent	-	ID + EP or IM	Naked or EP	-
	[105]	*Hepacivirus*	GBV-B - HCV	Hepatitis virus	RNA	Transcript	Uncapped RNA	pT7	-	Callithrix jacchus	NHP	-	Intrahepatic	Naked	-
	[106]	*Orthohepevirus*	HEV	Hepatitis virus	RNA	Transcript	Capped RNA	pT7		Rat	Rodent	-	Intrahepatic	Naked	-
	[107]	*Alphavirus*	CHIKV	Arbovirus	DNA	Cloned	Plasmid	pCMV	HDVr	Mice	Rodent	-	IM + EP	EP	-
	[108]	*Flavivirus*	YFV	Arbovirus	DNA	Cloned	Plasmid	pCMV	-	Mice	Rodent	-	IM + EP	EP	-
2015	[109]	*Orthohepadnavirus*	HBV	Hepatitis virus	DNA	Cloned	Plasmid	-	-	Mice	Rodent	-	IV (hydrodynamic)	Hydrodynamic	-
	[110]	*Flavivirus*	YFV	Arbovirus	DNA	Cloned	BAC	pCMV	HDVr	Transgenic mice	Rodent	AAD or A129 or 129	IM + EP	EP	NR
	[111]	*Orthohepadnavirus*	HBV	Hepatitis virus	DNA	Cloned	Plasmid	-	-	Mice	Rodent	-	IV (hydrodynamic)	Hydrodynamic	-
	[112]	*Circovirus*	DuCV	Veterinary virus	DNA	Cloned	Plasmid	Eukaryotic promoter	-	Duck	Bird	-	IM	Cationic lipids	"Lipofectamine 2000" kit
	[113]	*Orthohepevirus*	HEV	Hepatitis virus	RNA	Transcript	Capped RNA	pT7		Transgenic rat	Rodent	Nude	Intrahepatic	Naked	-
	[114]	*Aphtovirus*	FMDV	Veterinary virus	DNA	Cloned	Plasmid	Several	Several	Mice	Rodent	-	IP	Cationic lipids	"Lipofectamine 2000" kit
	[115]	*Orthohepevirus*	HEV	Hepatitis virus	RNA	Transcript	Capped RNA	pT7		Chicken	Bird	-	Intrahepatic	Naked	-

2015	[116]	*Circovirus*	PCV	Veterinary virus	DNA	Cloned	Plasmid	pCMV	-	Mice	Rodent	-	IM	Naked	-
	[117]	*Orthohepadnavirus*	HBV	Hepatitis virus	DNA	Cloned	Plasmid	-	-	Mice	Rodent	-	IV (hydrodynamic)	Hydrodynamic	-
2016	[118]	*Flavivirus*	WNV	Arbovirus	DNA	Cloned	Plasmid	pCMV	HDVr + BGHTT	Mice	Rodent	-	IM or ID (Gene gun)	Naked or Gene Gun	Gold nanoparticles
2017	[119]	*Flavivirus*	JEV	Arbovirus	DNA	Cloned	Plasmid	pCMV	HDVr	Mice	Rodent	-	IM + EP	EP	-
	[120]	*Flavivirus*	WNV	Arbovirus	DNA	Cloned	Plasmid	pCMV	HDVr + BGHTT	Mice	Rodent	-	IC	Naked	-
2018	[121]	*Flavivirus*	ZIKV	Arbovirus	DNA	Cloned	Plasmid	pSV40	HDVr + SV40-poly(A)	Transgenic mice	Rodent	A129	IM or IM+EP	Naked or EP	-
2019	[122]	*Flavivirus*	YFV	Arbovirus	DNA	Cloned	BAC	pSV40	-	Transgenic mice	Rodent	AG129	IP	Calcium meditated	Calcium microflowers
	[123]	*Alphavirus*	VEEV	Arbovirus	DNA	Cloned	Plasmid	pCMV	-	Mice	Rodent	-	IM + EP	EP	-
2020	[124]	*Flavivirus*	ZIKV	Arbovirus	DNA	Cloned	BAC	pCMV	HDVr + BGHTT	Transgenic mice	Rodent	A129	SC or IM or IP	Cationic lipids or Cationic lipids+Cationic polymers	"Lipofectamine 2000" kit or "GenJet" kit
2021	[125]	*Flavivirus*	DENV ZIKV	Arbovirus	DNA	Cloned	Plasmid	pCMV	-	Transgenic mice	Rodent	AG129	IP	Cationic polymers	"in vivo Jet-PEI" kit
2022	[126]	*Aphtovirus*	FMDV	Veterinary virus	RNA	Purified	-	-	-	Cow	Other	-	Intralinguale	Naked	-
	[127]	*Alphavirus*	VEEV	Arbovirus	DNA	Cloned	Plasmid	pCMV	-	Rabbit	Rabbit	-	ID	Naked	-
	[128]	*Orthohepevirus*	HEV	Hepatitis virus	RNA	Transcript	Capped or uncapped RNA	pT7	-	Mongolian gerbil	Rodent	-	Intrahepatic	Naked	-
2024	[129]	*Flavivirus*	TBEV	Arbovirus	DNA	Cloned	DNA fragments	pCMV	HDVr + SV40-poly(A)	Mice	Rodent	-	IM or IM+EP	Naked or EP	-
		Flavivirus	JEV	Arbovirus	DNA	Cloned	DNA fragments	pCMV	HDVr + SV40-poly(A)	Mice	Rodent	-	IM+EP	EP	-
		Alphavirus	CHIKV	Arbovirus	DNA	Cloned	DNA fragments	pCMV	HDVr + SV40-poly(A)	Mice	Rodent	-	IM+EP	EP	-
	[130]	*Enterovirus*	EV-A71	Enterovirus	DNA	Cloned	Plamid	pSV40 or pCMV	HDVr + SV40-poly(A)	Mice	Rodent	BALB/C or A129	IC or IM+EP	EP	-

List of abbreviations

AtGHV : Ateline gammaherpersvirus

BLV : Bovine leukemia virus

BGHTT : Bovine growth hormone transcriptional terminator

CAEV : Caprine arthritis encephalitis virus

CHIKV : Chikungunya virus

CMV : Cytomegalovirus

DEAE : Diethylaminoethyl

DENV : Dengue virus

DHBV : Duck hepatitis B virus

DuCV : Duck circovirus

EEEV : Eastern equine encephalitis virus

EMCV : Encephalomyocarditis virus

EV-A71 : Enterovirus 71

FELV : Feline leukemia virus

FIV : Feline immunodeficiency virus

FMDV : Foot-and-mouth disease virus

GBV-B : G.B. virus B

GSHV : Ground squirrel hepatitis virus

HAV : Hepatitis A virus

HBV : Hepatitis B virus

HCV : Hepatitis C virus

HDV : Hepatitis D virus

HDVr : Hepatitis D virus ribozyme

HEV : Hepatitis E virus

HIV : Human immunodeficiency virus

HTLV : Human T-cell leukemia virus

ID : Intradermal

IM : Intramuscular

IP : Intraperitoneal

ITV : Israel turkey meningoencephalitis virus

IV : Intravenous

JEV : Japanese encephalitis virus

KPV2 : Kappapapillomavirus 2

LDV : Lactate dehydrogenase-elevating Virus

MuLV : Murine leukemia virus

MuPyV : Murine polyamovirus

MVEV : Murray Valley encephalitis virus

NHP : Non-human primate

pCMV : CMV promoter

PCV: Porcine circovirus

PV : Poliovirus

PRRSV : Porcine reproductive and respiratory syndrome virus

Poly(a) : polyadenylation signal

pSV40 : SV40 promoter

PyV : Polyomavirus

RHDV : Rabbit hemorrhagic disease virus

SAdV : Simian adenovirus

SaGHV : Saimiri gammaherpersvirus

SC : Subcutaneous

SIV : Simian immunodeficiency virus

SV40 : Simian virus 40

TBEV : Tick-borne encephalitis virus

TMEV : Theiler's encephalomyelitis virus

TTSV : Torque teno virus

VEEV : Venezuelan equine encephalitis virus

WNV : West Nile virus

YFV : Yellow fever virus

ZIKV : Zika virus

α-CoV-1 : α-coronavirus

References

1. Colter, J.S., H.H. Bird, and R.A. Brown, *Infectivity of ribonucleic acid from Ehrlich ascites tumour cells infected with Mengo encephalitis.* Nature, 1957. **179**(4565): p. 859-60.
2. Colter, J.S., et al., *Infectivity of ribonucleic acid isolated from virus-infected tissues.* Virology, 1957. **4**(3): p. 522-32.
3. Wecker, E. and W. Schäfer, *Notizen: Eine infektiöse Komponente von Ribonucleinsäure-Charakter aus dem Virus der amerikanischen Pferde-Encephalomyelitis (Typ Ost).* Zeitschrift für Naturforschung B, 1957. **12**(6): p. 415-417.
4. Brown, F., R.F. Sellers, and D.L. Stewart, *Infectivity of ribonucleic acid from mice and tissue culture infected with the virus of foot-and-mouth disease.* Nature, 1958. **182**(4634): p. 535-6.
5. Ada, G.L. and S.G. Anderson, *Yield of infective ribonucleic acid from impure Murray Valley encephalitis virus after different treatments.* Nature, 1959. **183**(4664): p. 799-800.
6. Franklin, R.M., E. Wecker, and C. Henry, *C Some properties of an infectious ribonucleic acid from mouse encephalomyelitis virus.* Virology, 1959. **7**(2): p. 220-35.
7. Holland, J.J., L.L. Mc, and J.T. Syverton, *The mammalian cell-virus relationship. IV. Infection of naturally insusceptible cells with enterovirus ribonucleic acid.* J Exp Med, 1959. **110**(1): p. 65-80.
8. Holland, J.J., L.L. Mc, and J.T. Syverton, *Mammalian cell-virus relationship. III. Poliovirus production by non-primate cells exposed to poliovirus ribonucleic acid.* Proc Soc Exp Biol Med, 1959. **100**(4): p. 843-5.
9. Sokol, F., H. Libikova, and J. Zemla, *Infectious ribonucleic acid from mouse brains infected with tick-borne encephalitis virus.* Nature, 1959. **184(Suppl 20)**(4698): p. 1581.
10. Spuhler, V., *[Infectiousness of ribonucleic acid fraction of foot & mouth disease virus].* Experientia, 1959. **15**(4): p. 155.
11. Thomas, J.A. and J. Leclerc, *[The infectious ribonucleic acid of foot-and-mouth disease virus].* C R Hebd Seances Acad Sci, 1959. **248**(4): p. 606-9.
12. Ito, Y., *A tumor-producing factor extracted by phenol from papillomatous tissue (Shope) of cottontail rabbits.* Virology, 1960. **12**(4): p. 596-601.
13. Harris, R.J., F.C. Chesterman, and G. Negroni, *Induction of tumours in newborn ferrets with Mill Hill polyoma virus.* Lancet, 1961. **1**(7181): p. 788-91.
14. Ito, Y. and C.A. Evans, *Induction of Tumors in Domestic Rabbits with Nucleic Acid Preparations from Partially Purified Shope Papilloma Virus and from Extracts of the Papillomas of Domestic and Cottontail Rabbits.* J Exp Med, 1961. **114**(4): p. 485-500.
15. Nakamura, M., *Infectious ribonucleic acids derived from mouse brains infected with two kinds of arbor virus group B.* Nature, 1961. **191**(4788): p. 624.
16. Notkins, A.L. and C. Scheele, *An Infectious Nucleic Acid from the Lactic Dehydrogenase Agent.* Virology, 1963. **20**(4): p. 640-2.
17. Notkins, A.L., *Recovery of an Infectious Ribonucleic Acid from the Lactic Dehydrogenase Agent by Treatment with Ether.* Virology, 1964. **22**(4): p. 563-7.
18. Orth, G., et al., *Infectious and Oncogenic Effect of DNA Extracted from Cells Infected with Polyoma Virus.* Proc Soc Exp Biol Med, 1964. **115**(4): p. 1090-5.
19. Notkins, A.L., *Recovery of an infectious ribonucleic acid from the lactic dehydrogenase virus following extraction with butanol or chloroform.* Biochim Biophys Acta, 1965. **103**(3): p. 509-11.
20. Youngner, J.S. and M.E. Kelly, *Inhibition by Exogenous Interferon of Replication of Poliovirus Ribonucleic Acid in Chick Brain.* J Bacteriol, 1965. **90**(2): p. 443-5.
21. Akers, T.G., et al., *Virulence and Immunogenicity in Mice of Airborne Encephalomyocarditis Viruses and Their Infectious Nucleic Acids.* The Journal of Immunology, 1966. **97**(3): p. 379-385.
22. Akers, T.G., S.H. Madin, and F.L. Schaffer, *The Pathogenicity in Mice of Aerosols of Encephalomyocarditis Group Viruses or Their Infectious Nucleic Acids.* The Journal of Immunology, 1968. **100**(1): p. 120-127.
23. Burnett, J.P. and J.A. Harrington, *Simian adenovirus SA7 DNA: chemical, physical, and biological studies.* Proc Natl Acad Sci U S A, 1968. **60**(3): p. 1023-9.
24. Norman, J.O., A.W. McClurkin, and H.L. Bachrach, *Infectious nucleic acid from a transmissible agent causing gastroenteritis in pigs.* J Comp Pathol, 1968. **78**(2): p. 227-35.
25. Nir, Y., *Preparation of an infectious ribonucleic acid derived from mouse brains infected with Israel turkey meningo-encephalitis virus.* Arch Gesamte Virusforsch, 1970. **29**(2): p. 215-21.
26. Mayne, N., J.P. Burnett, and L.K. Butler, *Tumour induction by simian adenovirus SA7 DNA fragments.* Nat New Biol, 1971. **232**(2): p. 182-3.
27. Sol, C.J.A. and J. van der Noordaa, *Oncogenicity of SV40 DNA in the Syrian Hamster.* Journal of General Virology, 1977. **37**(3): p. 635-638.

28. Fleckenstein, B., et al., *Tumour induction with DNA of oncogenic primate herpesviruses.* Nature, 1978. **274**(5666): p. 57-9.
29. Israel, M.A., et al., *Biological activity of polyoma viral DNA in mice and hamsters.* J Virol, 1979. **29**(3): p. 990-6.
30. Will, H., et al., *Cloned HBV DNA causes hepatitis in chimpanzees.* Nature, 1982. **299**(5885): p. 740-2.
31. Dubensky, T.W., B.A. Campbell, and L.P. Villarreal, *Direct transfection of viral and plasmid DNA into the liver or spleen of mice.* Proc Natl Acad Sci U S A, 1984. **81**(23): p. 7529-33.
32. Seeger, C., D. Ganem, and H.E. Varmus, *The cloned genome of ground squirrel hepatitis virus is infectious in the animal.* Proc Natl Acad Sci U S A, 1984. **81**(18): p. 5849-52.
33. Sprengel, R., et al., *Cloned duck hepatitis B virus DNA is infectious in Pekin ducks.* J Virol, 1984. **52**(3): p. 932-7.
34. Will, H., et al., *Infectious hepatitis B virus from cloned DNA of known nucleotide sequence.* Proc Natl Acad Sci U S A, 1985. **82**(3): p. 891-5.
35. Sprengel, R., H.E. Varmus, and D. Ganem, *Homologous recombination between hepadnaviral genomes following in vivo DNA transfection: implications for studies of viral infectivity.* Virology, 1987. **159**(2): p. 454-6.
36. Sureau, C., et al., *Cloned hepatitis delta virus cDNA is infectious in the chimpanzee.* J Virol, 1989. **63**(10): p. 4292-7.
37. Ohlinger, V.F., et al., *Identification and characterization of the virus causing rabbit hemorrhagic disease.* J Virol, 1990. **64**(7): p. 3331-6.
38. Letvin, N.L., et al., *Risks of handling HIV.* Nature, 1991. **349**(6310): p. 573.
39. Emerson, S.U., et al., *cDNA clone of hepatitis A virus encoding a virulent virus: induction of viral hepatitis by direct nucleic acid transfection of marmosets.* J Virol, 1992. **66**(11): p. 6649-54.
40. Portis, J.L., F.J. McAtee, and S.C. Kayman, *Infectivity of retroviral DNA in vivo.* J Acquir Immune Defic Syndr (1988), 1992. **5**(12): p. 1272-3.
41. Willems, L., et al., *In vivo transfection of bovine leukemia provirus into sheep.* Virology, 1992. **189**(2): p. 775-7.
42. Willems, L., et al., *In vivo infection of sheep by bovine leukemia virus mutants.* J Virol, 1993. **67**(7): p. 4078-85.
43. Willems, L., et al., *Attenuation of bovine leukemia virus by deletion of R3 and G4 open reading frames.* Proc Natl Acad Sci U S A, 1994. **91**(24): p. 11532-6.
44. Harmache, A., et al., *The caprine arthritis encephalitis virus tat gene is dispensable for efficient viral replication in vitro and in vivo.* J Virol, 1995. **69**(9): p. 5445-54.
45. Polo, J.M., et al., *Replication of hepatitis delta virus RNA in mice after intramuscular injection of plasmid DNA.* J Virol, 1995. **69**(8): p. 5203-7.
46. Shaffer, D.R., et al., *A hepatitis A virus deletion mutant which lacks the first pyrimidine-rich tract of the 5' nontranslated RNA remains virulent in primates after direct intrahepatic nucleic acid transfection.* J Virol, 1995. **69**(10): p. 6600-4.
47. Zhao, T.M., et al., *Infectivity of chimeric human T-cell leukemia virus type I molecular clones assessed by naked DNA inoculation.* Proc Natl Acad Sci U S A, 1996. **93**(13): p. 6653-8.
48. Boris-Lawrie, K., et al., *In vivo study of genetically simplified bovine leukemia virus derivatives that lack tax and rex.* J Virol, 1997. **71**(2): p. 1514-20.
49. Kolykhalov, A.A., et al., *Transmission of hepatitis C by intrahepatic inoculation with transcribed RNA.* Science, 1997. **277**(5325): p. 570-4.
50. Rigby, M.A., et al., *Comparative efficiency of feline immunodeficiency virus infection by DNA inoculation.* AIDS Res Hum Retroviruses, 1997. **13**(5): p. 405-12.
51. Sparger, E.E., et al., *Infection of cats by injection with DNA of a feline immunodeficiency virus molecular clone.* Virology, 1997. **238**(1): p. 157-60.
52. Ward, G., E. Rieder, and P.W. Mason, *Plasmid DNA encoding replicating foot-and-mouth disease virus genomes induces antiviral immune responses in swine.* J Virol, 1997. **71**(10): p. 7442-7.
53. Yanagi, M., et al., *Transcripts from a single full-length cDNA clone of hepatitis C virus are infectious when directly transfected into the liver of a chimpanzee.* Proc Natl Acad Sci U S A, 1997. **94**(16): p. 8738-43.
54. Chen, H., et al., *Pathogenicity induced by feline leukemia virus, Rickard strain, subgroup A plasmid DNA (pFRA).* J Virol, 1998. **72**(9): p. 7048-56.
55. Harmache, A., et al., *Priming with tat-deleted caprine arthritis encephalitis virus (CAEV) proviral DNA or live virus protects goats from challenge with pathogenic CAEV.* J Virol, 1998. **72**(8): p. 6796-804.

56. Beard, C., et al., *Development of DNA vaccines for foot-and-mouth disease, evaluation of vaccines encoding replicating and non-replicating nucleic acids in swine.* J Biotechnol, 1999. **73**(2-3): p. 243-9.
57. Beard, M.R., et al., *An infectious molecular clone of a Japanese genotype 1b hepatitis C virus.* Hepatology, 1999. **30**(1): p. 316-24.
58. Bukh, J., C.L. Apgar, and M. Yanagi, *Toward a surrogate model for hepatitis C virus: An infectious molecular clone of the GB virus-B hepatitis agent.* Virology, 1999. **262**(2): p. 470-8.
59. Hong, Z., et al., *Generation of transmissible hepatitis C virions from a molecular clone in chimpanzees.* Virology, 1999. **256**(1): p. 36-44.
60. Kucerova, L., et al., *Bovine leukemia virus structural gene vectors are immunogenic and lack pathogenicity in a rabbit model.* J Virol, 1999. **73**(10): p. 8160-6.
61. Liska, V., et al., *Viremia and AIDS in rhesus macaques after intramuscular inoculation of plasmid DNA encoding full-length SIVmac239.* AIDS Res Hum Retroviruses, 1999. **15**(5): p. 445-50.
62. Major, M.E., et al., *Long-term follow-up of chimpanzees inoculated with the first infectious clone for hepatitis C virus.* J Virol, 1999. **73**(4): p. 3317-25.
63. Yanagi, M., et al., *In vivo analysis of the 3' untranslated region of the hepatitis C virus after in vitro mutagenesis of an infectious cDNA clone.* Proc Natl Acad Sci U S A, 1999. **96**(5): p. 2291-5.
64. Forns, X., et al., *Hepatitis C virus lacking the hypervariable region 1 of the second envelope protein is infectious and causes acute resolving or persistent infection in chimpanzees.* Proc Natl Acad Sci U S A, 2000. **97**(24): p. 13318-23.
65. Kolykhalov, A.A., et al., *Hepatitis C virus-encoded enzymatic activities and conserved RNA elements in the 3' nontranslated region are essential for virus replication in vivo.* J Virol, 2000. **74**(4): p. 2046-51.
66. Phipps, A.J., et al., *Neurophysiologic and immunologic abnormalities associated with feline immunodeficiency virus molecular clone FIV-PPR DNA inoculation.* J Acquir Immune Defic Syndr, 2000. **23**(1): p. 8-16.
67. Emerson, S.U., et al., *Recombinant hepatitis E virus genomes infectious for primates: importance of capping and discovery of a cis-reactive element.* Proc Natl Acad Sci U S A, 2001. **98**(26): p. 15270-5.
68. Kent, S.J., et al., *Vaccination with attenuated simian immunodeficiency virus by DNA inoculation.* J Virol, 2001. **75**(23): p. 11930-4.
69. Lanford, R.E., et al., *Infectious cDNA clone of the hepatitis C virus genotype 1 prototype sequence.* J Gen Virol, 2001. **82**(Pt 6): p. 1291-1297.
70. Merezak, C., et al., *Suboptimal enhancer sequences are required for efficient bovine leukemia virus propagation in vivo: implications for viral latency.* J Virol, 2001. **75**(15): p. 6977-88.
71. Pion, M., et al., *Extensively deleted simian immunodeficiency virus (SIV) DNA in macaques inoculated with supercoiled plasmid DNA encoding full-length SIVmac239.* Virology, 2001. **289**(1): p. 103-13.
72. Reichert, M., et al., *Role of the proline-rich motif of bovine leukemia virus transmembrane protein gp30 in viral load and pathogenicity in sheep.* J Virol, 2001. **75**(17): p. 8082-9.
73. Bukh, J., et al., *Mutations that permit efficient replication of hepatitis C virus RNA in Huh-7 cells prevent productive replication in chimpanzees.* Proc Natl Acad Sci U S A, 2002. **99**(22): p. 14416-21.
74. Fenaux, M., et al., *Cloned genomic DNA of type 2 porcine circovirus is infectious when injected directly into the liver and lymph nodes of pigs: characterization of clinical disease, virus distribution, and pathologic lesions.* J Virol, 2002. **76**(2): p. 541-51.
75. Baranowski, E., et al., *Recovery of infectious foot-and-mouth disease virus from suckling mice after direct inoculation with in vitro-transcribed RNA.* J Virol, 2003. **77**(20): p. 11290-5.
76. Fenaux, M., et al., *Immunogenicity and pathogenicity of chimeric infectious DNA clones of pathogenic porcine circovirus type 2 (PCV2) and nonpathogenic PCV1 in weanling pigs.* J Virol, 2003. **77**(20): p. 11232-43.
77. Hall, R.A., et al., *DNA vaccine coding for the full-length infectious Kunjin virus RNA protects mice against the New York strain of West Nile virus.* Proc Natl Acad Sci U S A, 2003. **100**(18): p. 10460-4.
78. Pion, M., et al., *Truncated forms of human and simian immunodeficiency virus in infected individuals and rhesus macaques are unique or rare quasispecies.* Virology, 2003. **311**(1): p. 157-68.
79. Sakai, A., et al., *The p7 polypeptide of hepatitis C virus is critical for infectivity and contains functionally important genotype-specific sequences.* Proc Natl Acad Sci U S A, 2003. **100**(20): p. 11646-51.
80. Maeda, N., et al., *Hepatitis C virus infection in human liver tissue engrafted in mice with an infectious molecular clone.* Liver Int, 2004. **24**(3): p. 259-67.
81. Nam, J.H., et al., *In vivo analysis of the 3' untranslated region of GB virus B after in vitro mutagenesis of an infectious cDNA clone: persistent infection in a transfected tamarin.* J Virol, 2004. **78**(17): p. 9389-99.
82. Roca, M., et al., *In vitro and in vivo characterization of an infectious clone of a European strain of porcine circovirus type 2.* J Gen Virol, 2004. **85**(Pt 5): p. 1259-1266.

83. Huang, F.F., et al., *Construction and characterization of infectious cDNA clones of a chicken strain of hepatitis E virus (HEV), avian HEV.* J Gen Virol, 2005. **86**(Pt 9): p. 2585-2593.
84. Huang, Y.W., et al., *Capped RNA transcripts of full-length cDNA clones of swine hepatitis E virus are replication competent when transfected into Huh7 cells and infectious when intrahepatically inoculated into pigs.* J Virol, 2005. **79**(3): p. 1552-8.
85. Lee, Y.R., et al., *Suckling mice were used to detect infectious dengue-2 viruses by intracerebral injection of the full-length RNA transcript.* Intervirology, 2005. **48**(2-3): p. 161-6.
86. Liu, G., et al., *Recovery of infectious rabbit hemorrhagic disease virus from rabbits after direct inoculation with in vitro-transcribed RNA.* J Virol, 2006. **80**(13): p. 6597-602.
87. Seregin, A., et al., *Immunogenicity of West Nile virus infectious DNA and its noninfectious derivatives.* Virology, 2006. **356**(1-2): p. 115-25.
88. Haqshenas, G., et al., *A chimeric GB virus B encoding the hepatitis C virus hypervariable region 1 is infectious in vivo.* J Gen Virol, 2007. **88**(Pt 3): p. 895-902.
89. Huang, Y.W., et al., *Initiation at the third in-frame AUG codon of open reading frame 3 of the hepatitis E virus is essential for viral infectivity in vivo.* J Virol, 2007. **81**(6): p. 3018-26.
90. Key, K.F., et al., *Direct inoculation of RNA transcripts from an infectious cDNA clone of porcine reproductive and respiratory syndrome virus (PRRSV) into the lymph nodes and tonsils of pigs initiates PRRSV infection in vivo.* Arch Virol, 2007. **152**(7): p. 1383-7.
91. Liu, F.J., et al., *Establishment and primary application of a mouse model with hepatitis B virus replication.* World J Gastroenterol, 2007. **13**(40): p. 5324-30.
92. Sakai, A., et al., *In vivo study of the HC-TN strain of hepatitis C virus recovered from a patient with fulminant hepatitis: RNA transcripts of a molecular clone (pHC-TN) are infectious in chimpanzees but not in Huh7.5 cells.* J Virol, 2007. **81**(13): p. 7208-19.
93. Pudupakam, R.S., et al., *Deletions of the hypervariable region (HVR) in open reading frame 1 of hepatitis E virus do not abolish virus infectivity: evidence for attenuation of HVR deletion mutants in vivo.* J Virol, 2009. **83**(1): p. 384-95.
94. Rodriguez Pulido, M., et al., *Attenuated foot-and-mouth disease virus RNA carrying a deletion in the 3' noncoding region can elicit immunity in swine.* J Virol, 2009. **83**(8): p. 3475-85.
95. Gottwein, J.M., et al., *Novel infectious cDNA clones of hepatitis C virus genotype 3a (strain S52) and 4a (strain ED43): genetic analyses and in vivo pathogenesis studies.* J Virol, 2010. **84**(10): p. 5277-93.
96. Cordoba, L., et al., *Three amino acid mutations (F51L, T59A, and S390L) in the capsid protein of the hepatitis E virus collectively contribute to virus attenuation.* J Virol, 2011. **85**(11): p. 5338-49.
97. Kwon, H.M., et al., *Construction of an infectious cDNA clone of avian hepatitis E virus (avian HEV) recovered from a clinically healthy chicken in the United States and characterization of its pathogenicity in specific-pathogen-free chickens.* Vet Microbiol, 2011. **147**(3-4): p. 310-9.
98. Cordoba, L., et al., *Rescue of a genotype 4 human hepatitis E virus from cloned cDNA and characterization of intergenotypic chimeric viruses in cultured human liver cells and in pigs.* J Gen Virol, 2012. **93**(Pt 10): p. 2183-2194.
99. He, F., et al., *Inhibition of hepatitis B Virus replication by hepatocyte nuclear factor 4-alpha specific short hairpin RNA.* Liver Int, 2012. **32**(5): p. 742-51.
100. Huang, Y.W., et al., *Rescue of a porcine anellovirus (torque teno sus virus 2) from cloned genomic DNA in pigs.* J Virol, 2012. **86**(11): p. 6042-54.
101. Liu, F.J., et al., *Functional Characterization of Interferon Regulation Element of Hepatitis B virus Genome In Vivo.* Indian J Virol, 2012. **23**(3): p. 278-85.
102. Tretyakova, I., et al., *Novel vaccine against Venezuelan equine encephalitis combines advantages of DNA immunization and a live attenuated vaccine.* Vaccine, 2013. **31**(7): p. 1019-25.
103. Zhu, Y., et al., *Infectivity of a genotype 4 hepatitis E virus cDNA clone by intrahepatic inoculation of laboratory rats.* Vet Microbiol, 2013. **166**(3-4): p. 405-11.
104. Hallengard, D., et al., *Novel attenuated Chikungunya vaccine candidates elicit protective immunity in C57BL/6 mice.* J Virol, 2014. **88**(5): p. 2858-66.
105. Li, T., et al., *Infection of common marmosets with hepatitis C virus/GB virus-B chimeras.* Hepatology, 2014. **59**(3): p. 789-802.
106. Si, F., et al., *Construction of an infectious cDNA clone of a swine genotype 3 HEV strain isolated in Shanghai, China.* Intervirology, 2014. **57**(2): p. 74-82.
107. Tretyakova, I., et al., *DNA vaccine initiates replication of live attenuated chikungunya virus in vitro and elicits protective immune response in mice.* J Infect Dis, 2014. **209**(12): p. 1882-90.

108. Tretyakova, I., et al., *Plasmid DNA initiates replication of yellow fever vaccine in vitro and elicits virus-specific immune response in mice.* Virology, 2014. **468-470**: p. 28-35.
109. Chen, E.Q., et al., *The efficacy of zinc finger antiviral protein against hepatitis B virus transcription and replication in tansgenic mouse model.* Virol J, 2015. **12**(1): p. 25.
110. Jiang, X., et al., *Molecular and immunological characterization of a DNA-launched yellow fever virus 17D infectious clone.* J Gen Virol, 2015. **96**(Pt 4): p. 804-814.
111. Leng, X.H., et al., *Biological characteristics of the A1762T/G1764A mutant strain of hepatitis B virus in vivo.* Mol Med Rep, 2015. **12**(4): p. 5141-8.
112. Li, P., et al., *Rescue of a duck circovirus from an infectious DNA clone in ducklings.* Virol J, 2015. **12**(1): p. 82.
113. Li, T.C., et al., *Construction and characterization of an infectious cDNA clone of rat hepatitis E virus.* J Gen Virol, 2015. **96**(Pt 6): p. 1320-1327.
114. Lian, K., et al., *Recovery of infectious type Asia1 foot-and-mouth disease virus from suckling mice directly inoculated with an RNA polymerase I/II-driven unidirectional transcription plasmid.* Virus Res, 2015. **208**: p. 73-81.
115. Park, S.J., et al., *Construction of an infectious cDNA clone of genotype 1 avian hepatitis E virus: characterization of its pathogenicity in broiler breeders and demonstration of its utility in studying the role of the hypervariable region in virus replication.* J Gen Virol, 2015. **96**(Pt 5): p. 1015-1026.
116. Wang, W.C., et al., *Construction of a novel porcine circovirus type 2 infectious clone as a basis for the development of a PCV2 iDNA vaccine.* J Virol Methods, 2015. **220**: p. 21-6.
117. Zhou, Q., et al., *RPB5-Mediating Protein Suppresses Hepatitis B Virus (HBV) Transcription and Replication by Counteracting the Transcriptional Activation of Hepatitis B virus X Protein in HBV Replication Mouse Model.* Jundishapur J Microbiol, 2015. **8**(9): p. e21936.
118. Yamshchikov, V., M. Manuvakhova, and E. Rodriguez, *Development of a human live attenuated West Nile infectious DNA vaccine: Suitability of attenuating mutations found in SA14-14-2 for WN vaccine design.* Virology, 2016. **487**: p. 198-206.
119. Nickols, B., et al., *Plasmid DNA launches live-attenuated Japanese encephalitis virus and elicits virus-neutralizing antibodies in BALB/c mice.* Virology, 2017. **512**: p. 66-73.
120. Yamshchikov, V., et al., *Development of a human live attenuated West Nile infectious DNA vaccine: Identification of a minimal mutation set conferring the attenuation level acceptable for a human vaccine.* Virology, 2017. **500**: p. 122-129.
121. Zou, J., et al., *A single-dose plasmid-launched live-attenuated Zika vaccine induces protective immunity.* EBioMedicine, 2018. **36**: p. 92-102.
122. Kum, D.B., et al., *Limited evolution of the yellow fever virus 17d in a mouse infection model.* Emerg Microbes Infect, 2019. **8**(1): p. 1734-1746.
123. Tretyakova, I., et al., *Novel DNA-launched Venezuelan equine encephalitis virus vaccine with rearranged genome.* Vaccine, 2019. **37**(25): p. 3317-3325.
124. Avila-Perez, G., et al., *In vivo rescue of recombinant Zika virus from an infectious cDNA clone and its implications in vaccine development.* Sci Rep, 2020. **10**(1): p. 512.
125. Chin, W.X., et al., *A single-dose live attenuated chimeric vaccine candidate against Zika virus.* NPJ Vaccines, 2021. **6**(1): p. 20.
126. Keck, H., et al., *Full-Length Genomic RNA of Foot-and-Mouth Disease Virus Is Infectious for Cattle by Injection.* Viruses, 2022. **14**(9): p. 1924.
127. Tretyakova, I., et al., *Live-Attenuated VEEV Vaccine Delivered by iDNA Using Microneedles Is Immunogenic in Rabbits.* Frontiers in Tropical Diseases, 2022. **3**.
128. Xu, L.D., et al., *Revisiting the Mongolian Gerbil Model for Hepatitis E Virus by Reverse Genetics.* Microbiol Spectr, 2022. **10**(2): p. e0219321.
129. Cochin, M., et al., *In vivo rescue of arboviruses directly from subgenomic DNA fragments.* Emerg Microbes Infect, 2024: p. 2356140.
130. Zhang, R.R., et al., *Rational design of a DNA-launched live attenuated vaccine against human enterovirus 71.* Virol Sin, 2024. **39**(5): p. 812-820.
